# Personalized medicine in common mental disorders

**DOI:** 10.1186/1878-5085-5-S1-A94

**Published:** 2014-02-11

**Authors:** Hans Jörgen Grabe

**Affiliations:** 1 Department of Psychiatry and Psychotherapy, Ellernholzstraße 1-2, 17475 Greifswald, Germany

## Scientific objectives

Mental disorders affect up to 40 % of all individuals living in Europe at least once a life time. Serious mental disorders with chronic or relapsing course like major depression bipolar disorder and schizophrenia are disabling and often disintegrating disorders. The early identification of subjects being at risk for serious mental disorders holds the promise of early intervention and better long-term outcomes. However, still reliable early indicators are largely missing to date. Like in dementia research major research initiatives need to be developed to address those urgent issues.

## Technological approaches

New MRI imaging technique, genotyping, gene methylation analyses, metabolom analyses, validated questionnaires for the assessment of psychosocial risk factors.

## Results interpretation

Advanced statistical modeling of brain imaging data, support vector machine approaches, systems integration of OMICS data; data mining approaches for risk marker identification.

## Outlook and expert recommendations

The identification of peripheral risk indicators of severe mental diseases is probably limited to rare but highly penetrative genetic variants. Possibly gene-methylation patterns that emerge in the light of abuse and traumatization be constitute another source of valuable information. The non-invasive MRI imaging technologies might be very promising but still time consuming and expensive. Especially functional MRI studies are only feasible in specialist centers. The most promising approach seems to create a set of specific markers throughout the different levels of information to integrate biological, psychosocial and structural information on each subject.

## References

[B1] Grabe HJ Schwahn C Appel K Mahler J Schulz A Spitzer C Barnow S John U Freyberger HJ Rosskopf D Völzke H Update on the 2005 paper: Moderation of mental and physical distress by polymorphisms in the 5-HT transporter gene by interacting with social stressors and chronic disease burden Mol Psychiatry 2011 16 4 354 6 10.1038/mp.2010.45 20386567

[B2] Appel K Schwahn C Mahler J Schulz A Spitzer C Fenske K Stender J Barnow S John U Teumer A Biffar R Nauck M Völzke H Freyberger HJ Grabe HJ Moderation of Adult Depression by a Polymorphism in the FKBP5 Gene and Childhood Physical Abuse in the General Population Neuropsychopharmacology 2011 36 10 1982 91 10.1038/npp.2011.81 21654733 PMC3158316

[B3] Grabe HJ Lange M Wolff B Völzke H Lucht M Freyberger HJ John U Cascorbi I Mental and physical distress is modulated by a polymorphism in the 5-HT transporter gene interacting with social stressors and chronic disease burden Mol Psychiatry 2005 10 220 4 10.1038/sj.mp.4001555 15263905

[B4] Stein JL Medland SE Vasquez AA Hibar DP Grabe HJ Meyer-Lindenberg A Morris DW Müller-Myhsok B Nichols TE Ophoff RA Paus T Pausova Z Penninx BW Potkin SG Sämann PG Saykin AJ Schumann G Smoller JW Wardlaw JM Weale ME Martin NG Franke B Wright MJ Thompson PM for the Enhancing NeuroImaging Genetics through Meta-Analysis (ENIGMA) Consortium Common genetic polymorphisms are associated with human hippocampal and intracranial volumes Nat Genet 2012 44 5 552 561 10.1038/ng.2250 22504417 PMC3635491

